# P-61. Prospective Analysis of Antimicrobial Therapy and Clinical Outcomes in Bacteremia across Consultation Approaches

**DOI:** 10.1093/ofid/ofaf695.290

**Published:** 2026-01-11

**Authors:** Sathya Narayanan, Suresh Kumar, Suhail Hassan Jalal, Rithik Dharan, Roshni Murali

**Affiliations:** Tamilnadu Dr.MGR University, Chennai, Tamil Nadu, India; Apollo Hospitals, Chennai, Tamil Nadu, India; The Tamil Nadu Dr. M.G.R. Medical University, Chennai, Tamil Nadu, India; The Tamil Nadu Dr. MGR Medical University, Chennai, Tamil Nadu, India; The Tamil Nadu Dr. M.G.R. Medical University, Chennai, Tamil Nadu, India

## Abstract

**Background:**

Bacteremia, a serious bloodstream infection, requires timely and precise antimicrobial therapy. In India,rising antibiotic resistance is largely due to misuse and overuse of antibiotics. Involvement of Infectious Diseases (ID) specialists is known to improve treatment optimality, yet evidence on the impact of adherence to ID recommendations is limited in Indian settings. This study evaluates clinical outcomes in inpatients with bacteremia, comparing those with followed, unfollowed, or no ID opinion, focusing on therapy duration, hospital stay, and severity scores to highlight stewardship practice.

**Figure:**
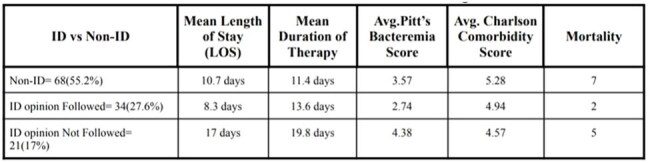
Clinical Outcomes Based on ID Involvement in Bacteremia Management

Table summarizing clinical outcomes in bacteremia cases stratified by ID consultation and adherence. Patients who followed ID recommendations had reduced length of stay, lower Pitt’s bacteremia scores, and reduced mortality, underscoring the clinical impact of ID-guided therapy.

**Figure:**
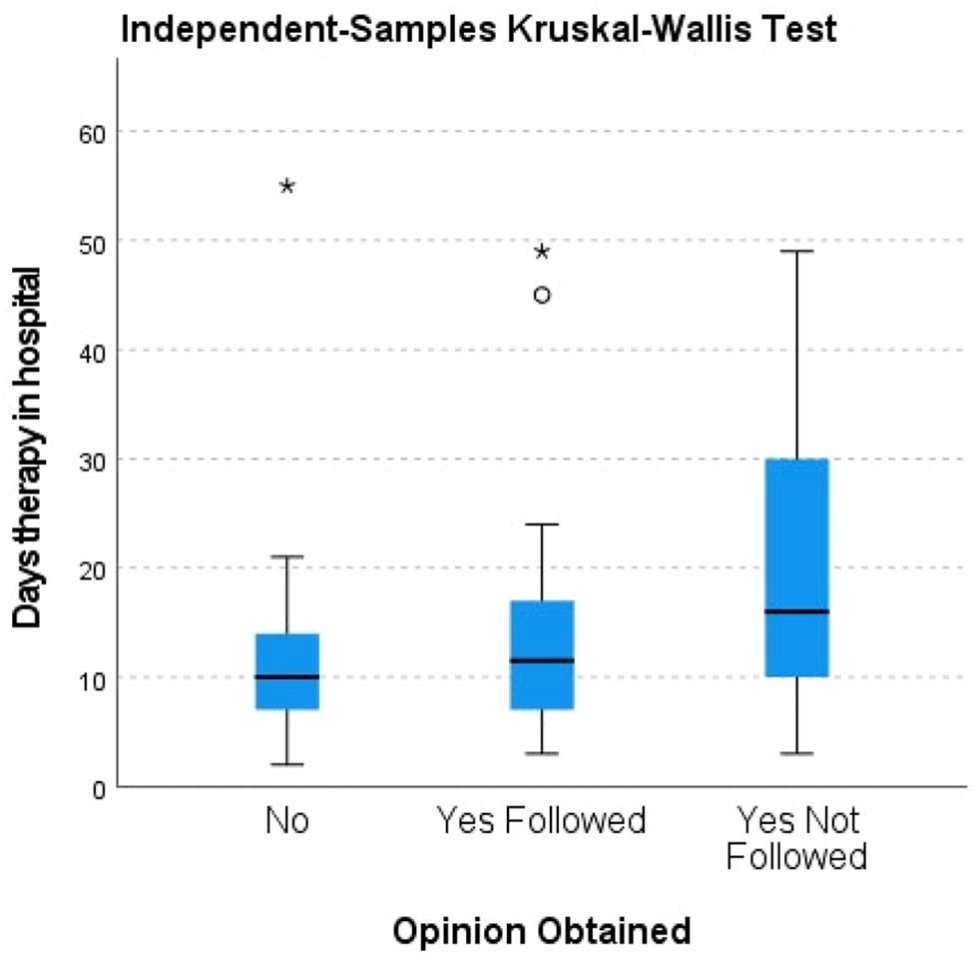
Effect of ID Opinion on Duration of Antimicrobial Therapy Boxplot showing therapy duration across three groups. Following ID opinion resulted in optimal antimicrobial duration, while non-adherence was associated with prolonged treatment.

**Methods:**

This prospective observational study was conducted from February to June 2024 at a South Indian tertiary care hospital. The Study Conducted in Inpatients department and included (n=130) with bloodstream infections, hospitalized >48 hours and receiving antimicrobial therapy. Patients categorized into three groups: no ID consult, ID opinion followed, and ID opinion not followed. Pitt’s bacteremia and Charlson Comorbidity scores were assessed. Length of stay and therapy duration were analyzed using Kruskal-Wallis; mortality and Pitt’s bacteremia score with Chi-square. Data were compiled in MS Excel and analyzed using SPSS v27.

**Figure:**
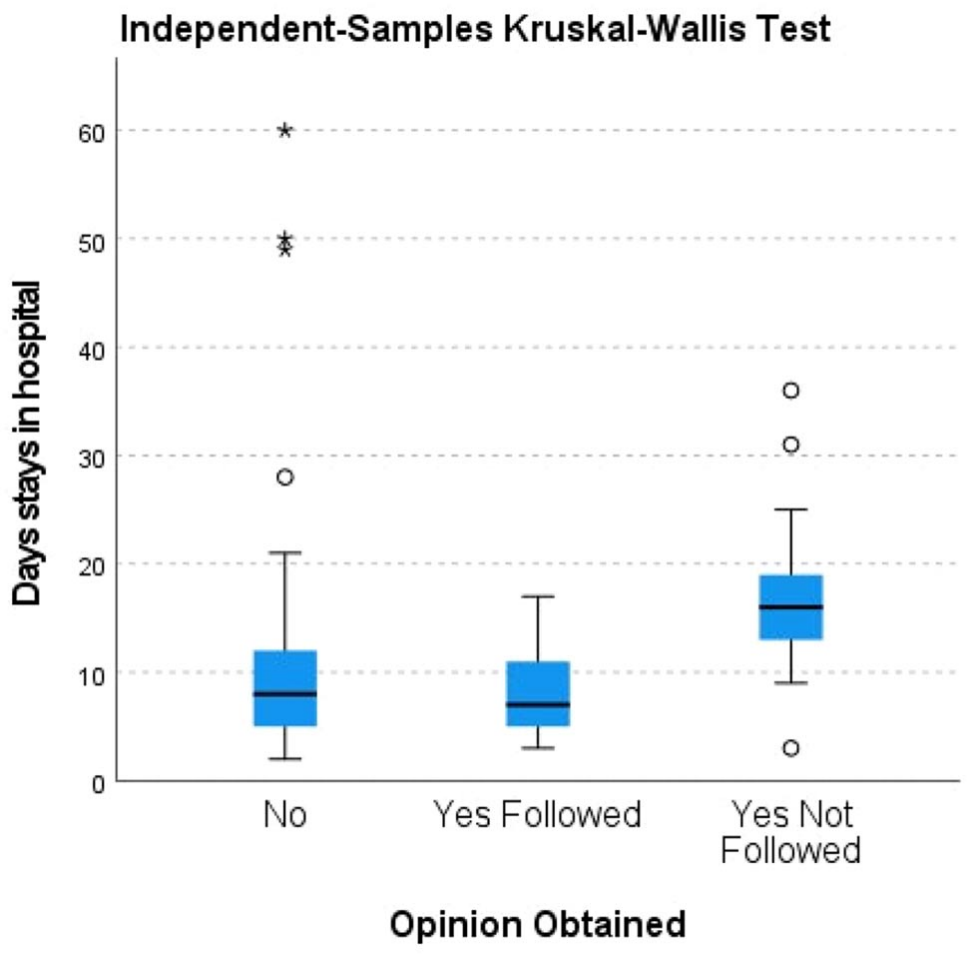
Impact of ID Consultation on Hospital Stay in Bacteremia Patients Boxplot representing hospital length of stay based on ID opinion status. Patients who received and followed ID specialist advice had significantly shorter hospital stays compared to those who did not receive or did not follow ID opinion.

**Figure:**
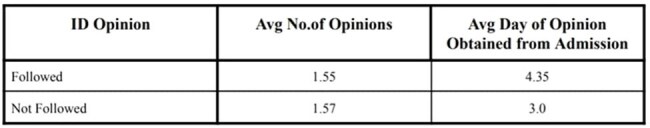
Timing and Frequency of ID Consultations: Comparison Between Followed and Unfollowed Opinions This table compares the average number and timing of Infectious Diseases (ID) consultations between cases where ID recommendations were followed versus not followed. While the average number of consultations was similar in both groups, ID input was sought earlier in patients whose advice was not followed.

**Results:**

Out of 871 Cultures Sent, 130(14.9%) were Positive with 7 excluded & 123 included. Patients without ID Consultation had mean Length of Stay(LOS)=10.7 and therapy duration=11.4 days. Those followed ID Opinion Shows Shorter LOS=8.3days(p< 0.001) and therapy duration=13.6 days. Not Followed Opinion Patients had Longer (LOS=17 days, therapy=19.8 days). Pitt scores in Followed Opinion & Not followed (2.74 vs 4.38, p< 0.001). No Mortality Difference were Observed (p=0.116).

**Conclusion:**

ID specialist involvement and adherence to their recommendations significantly improves bacteremia outcomes, reducing hospital stays and optimizing therapy duration, highlighting the critical role of specialist-guided antimicrobial management in bloodstream infections.

**Disclosures:**

All Authors: No reported disclosures

